# Multiphysics Study of Thermal Profiles and Residual Stress in Welding

**DOI:** 10.3390/ma17040886

**Published:** 2024-02-14

**Authors:** Yousung Han

**Affiliations:** Department of Mechatronics Engineering, Incheon National University, 119 Academy-ro, Yeonsu-gu, Incheon 22012, Republic of Korea; yshan@inu.ac.kr

**Keywords:** residual stress, finite element analysis, phase transformation, transformation plasticity

## Abstract

One of the effects of welding is residual stress. Welding involves complex tests concerning differences in values of the mechanical parameters of its regions as an effect of residual stress. Such multiphysics characteristics of welding pose a challenge in predicting residual stress. In the present study, a thermo-mechanical constitutive model considering phase transformation and transformation plasticity is implemented in the numerical model in ABAQUS user subroutines. In order to consider phase evolution in welding, the metallurgical parameters for Leblond’s phase equation were obtained from the calibration of DH36 steel with a CCT diagram. In addition, the effects of welding speed on thermal profiles and residual stress generation were investigated. Analysis has suggested that the width of the heat-affected zone (HAZ) decreases with an increase in welding speed, and the phase fraction is significantly affected by this kind of parameter. Such phase transformation has led to the generation of a compressive stress in the fusion zone (FZ) and HAZ. The volume difference between coexisting phases produces a compressive stress in cooling, and its magnitude was increased with martensite increasing.

## 1. Introduction

Welding involves complex material behaviors with respect to the interaction of mechanical, thermal, and metallurgical processes. The phase volume fraction of steel is determined by thermal profiles. Conversely, phase transformation leads to deformation due to volume dilation among the phases. In addition, during the welding process, the latent heat and heat capacity of a material influence temperature. Such multiphysical phenomena should be considered for more precise prediction of residual stress in a welded structure.

Iron atoms in steel are arranged in either a body-centered cubic (BCC) or face-centered cubic (FCC) structure, depending on temperature. The atomistic arrangement of ferritic steels (i.e., ferrite, bainite, or martensite) is a BCC structure at room temperature. However, when temperature exceeds the eutectoid temperature, steel has FCC structures and becomes austenite. During the heating process in welding, above the eutectoid temperature, the atomistic arrangement of the iron atoms is switched from BCC to FCC structure. Conversely, in the cooling process, austenitic steel transforms into ferrite, bainite, or martensite, depending on its cooling rate. In such phase transformations, plastic flows are generated even at the stress lower than the yield stress. This is known as transformation plasticity.

Many studies have been performed to understand the effects of phase transformation on microstructure evolution and material properties [1,2,3,4,5,6,7,8,9]. For example, Tsirkas et al. [10] developed the numerical model for laser welding simulations considering phase transformation using the Johnson–Mehl–Avrami law and the Koistinen–Marburger law. Rong et al. [1] examined the solid phase transformation in laser welding of EH36 steel. The effects of solid phase transformation on material properties, deformation, and residual stress were investigated. Lee and Chang [2] performed sequentially coupled thermomechanical simulations to study welding-induced residual stress in consideration of phase transformation. Most of these papers captured only phase transformation, and transformation plasticity was not explicitly considered. Leblond et al. [11,12] proposed a thermo-mechanical model incorporated with transformation plasticity. This constitutive model could predict thermal-induced residual stress more precisely.

A range of studies have been conducted to understand the influence of welding process parameters on mechanical properties, and thus the structural reliability of welded parts [13,14,15,16,17,18,19,20]. Ferro et al. [21] conducted FE simulations to examine the influence of different heat source models on thermal profiles in welding. Gannon et al. [22] performed FE simulations to examine how the welding sequence affects residual stress and warpage. Huang et al. [23] performed FE simulations to investigate the influence of the welding sequence on thermal-induced distortion, and proposed the optimal welding method to reduce buckling in the fabrication of thin plate. Biswas et al. [24] performed FE simulations to study the effects of the welding sequence on warpage and its magnitude in the welding of stiffened panels. Their results show that the welding sequence has great influence on the distortion of stiffened panel.

The influence of the heat source on residual stress was studied in several approaches [20,25,26,27,28,29,30,31,32,33]. Huang et al. [15] performed X-ray diffraction (XRD) analysis on welded structures to investigate the effects of strain hardening and annealing on the residual stress distribution of thin plates. Kong et al. [34] examined the suitability of double ellipsoidal and cylindrical heat source models for the numerical study of hybrid laser GMA welding. Benyounis et al. [35] conducted experimental studies to investigate the influence of welding parameters such as power, deposition speed, and focal position on the structural reliability of a welded structure to find the optimal welding conditions. The effects of welding speed on microstructural features and corrosion behavior were also investigated for the welding of AISI 201 stainless steel [36]. Microscopic analyses showed that the amount of grain coarsening decreases with an increase in welding speed. Zhang et al. [37] performed experimental analysis on the laser welding of stainless steel plate to investigate the effects of focal position and welding deposition speed on the bead geometry. The influence of welding speed on microstructural features were also analyzed for the welding of dissimilar aluminum and copper joints [38]. Analyses expressed that extremely high welding speed could suppress the formation of brittle compounds which produces a strong weld joint.

An implementation of thermomechanical constitutive equations to a numerical model has also been carried out to analyze deformation behaviors at high temperature. Gomes et al. [39] developed a numerical method to assess the safety design of a steel–timber connection subjected to both mechanical and thermal loadings. Bardel et al. [40] developed a numerical model with consideration of the phase transformation of aluminum alloy 6061 for a welding simulation. In their simulations, a metallurgical model was based on precipitate dissolution and coupled with thermomechanical constitutive equations. Rong et al. [1] developed a numerical model integrated with thermodynamic-based phase transformation to investigate residual stress generation in welding. Deng and Murakawa [41] developed a numerical model for welding simulation considering volume change due to martensite transformation, along with the thermomechanical behaviors of 9Cr-1Mo-alloy steel.

DH36 steel is a material widely used in the shipbuilding industry. It has high strength and good resistance against corrosion and fatigue at low temperatures. In welding, thermal profiles play a crucial role in the evolution of microstructure, and thus residual stress generation. In order to improve the reliability of welded components, it is necessary to understand the underlying mechanism of residual stress generation in welding. Among several welding process parameters, welding speed has a great influence on thermal profile and residual stress. Though several works have reported the effects of welding speed for different materials, the influence of welding speed on residual stress in DH36 steel have not been explicitly studied.

In the present study, a thermo-mechanical model incorporated with the constitutive equations of transformation plasticity was developed for welding simulations. In order to reflect the phase transformation during welding, the equation of phase evolution proposed by Leblond and Devaux [42] was implemented into the present numerical model, which was calibrated with a CCT diagram for the various cooling rates. The welding speed, arc voltage, and welding current were set to be 25–60 cm/min, 30.9 V, and 1170 A, respectively. The present numerical model allows the multiphysics simulation which is coupled with thermal, mechanical, and metallurgical behaviors of a material during welding. The numerical implementation was conducted using the ABAQUS user subroutines, UMAT, UMATHT, DFLUX, and UEXPAN. Some of the numerical studies take into account martensite transformation during welding (for example, refs. [2,43,44]). However, to author’s best knowledge, there are very few numerical studies in the literature on DH36 welding considering the phase transformation and transformation plasticity of all possible phases as the present study does. The results show that the lower welding speed leads to the higher peak temperature and slow cooling rate which result in the ferrite-dominant microstructures, while the phase fraction of martensite increases with the welding speed because of the fast cooling rate. It can be concluded that the welding speed has the significant effects on the thermal profiles and phase transformation, which lead to the generation of residual stress.

## 2. Methodology

### 2.1. Thermal-Metallurgical Analysis

In the present study, equations of the phase evolution proposed by Leblond and Devaux [42] were used to consider the phase transformation and transformation plasticity which is expressed as follows:

(1) $${\dot{p}}_{i}=-\sum_{j=1,j\neq i}^{4}\;A_{ij}\left(T,\dot{T}\right),i=1,2,\ldots ,4$$

where

(2) $$\sum_{i=1}^{4}\;p_{i}=1$$

In Equation (1), p is the phase volume proportion. The subscript i = 1, 2, 3, and 4 represents ferrite, bainite, martensite, and austenite, respectively. $A_{ij}(T,\dot{T})$, refers to the rate of the phase transformed from j to i. Note that $A_{ij}(T,\dot{T})$ is a function of temperature and temperature change rate. It is explained with more details in Section 2.2.

For martensitic transformation, the Koistinen–Marburger model [45] is used as Equation (3):

(3) $$\begin{array}{c} p_{3}(T)=1-exp\left\{a\left(T_{3,S}-T\right)\right\}\left(T\leq T_{3,S}\right)\\ with\;a=-\frac{\ln\left[p_{1}\left(T_{3,S}\right)+p_{2}\left(T_{3,S}\right)\right]}{T_{3,S}-T_{3,F}} \end{array}$$

Here, $T_{3,S}$ and $T_{3,F}$ are the start and finish temperatures for the martensite transformation. The energy balance equation and thermal boundary conditions are coupled together as in Equations (4)–(6). The phase proportions are obtained by solving Equation (1). Such results are plugged into Equation (4) for thermal analysis.

(4) $$\sum_{i}\;p_{i}(\rho c{)}_{i}\frac{dT}{dt}+\sum_{i}\;{\dot{p}}_{i}\rho_{i}H_{i}=\nabla \cdot \left(\sum_{i}\;p_{i}\lambda_{i}\nabla T\right)$$

(5) $$(-)\lambda \frac{\partial T}{\partial n}=q\;\mathrm{on}\;S_{q}$$

(6) $$(-)\lambda \frac{\partial T}{\partial n}=\chi_{1}\left(T-T_{0}\right)+\chi_{2}\left(T^{4}-T_{0}^{4}\right)\;\mathrm{on}\;S_{\theta }$$

Here, $\rho_{i}$ is the density; $c_{i}$ is specific heat capacity; $H_{i}$ is enthalpy; and $\lambda_{i}$ is thermal conductivity. $S_{q}$ represents the surface where the heat flux is applied; n is the normal vector to the surface; q is the heat flux from the heat source; $S_{\theta }$ represents the surface where the convection and radiation heat transfers occur. $T_{0}$ is the ambient temperature; and $\chi_{1}$ and $\chi_{2}$ are the convective coefficient and radiative coefficient, respectively.

In this work, the double ellipsoidal heat source model [46] is used for the welding simulations. In the model, the volumetric heat is applied in the form of Gaussian distribution as in Equations (7) and (8).

(7) $$Q_{f}=\frac{6\sqrt{3}f_{f}Q}{a_{f}bc\pi \sqrt{\pi }}exp\left(-\frac{3x^{2}}{a_{f}^{2}}-\frac{3y^{2}}{b^{2}}-\frac{3z^{2}}{c^{2}}\right)$$

(8) $$Q_{r}=\frac{6\sqrt{3}f_{r}Q}{a_{r}bc\pi \sqrt{\pi }}exp\left(-\frac{3x^{2}}{a_{r}^{2}}-\frac{3y^{2}}{b^{2}}-\frac{3z^{2}}{c^{2}}\right)$$

where $a_{f}$ and $a_{r}$ indicate the front and rear lengths of the heat source, respectively; and b and c indicate the width and depth of the heat source, respectively. $f_{f}$ and $f_{r}$ are parameters that assign the fraction of the heat applied in the front and rear parts, respectively. $Q_{f}\;$ and $Q_{r}$ are the heat energy of the front and rear parts in the double ellipsoidal heat source model, respectively. Q is the welding heat power. The values for the heat source parameters were chosen from the reference [47] and listed in Table 1.

### 2.2. Calibration with CCT Diagram

In the present study, the calibration with the CCT diagram [48] of DH36 steel was conducted for the precise calculation of the phase volume proportion. The term $A_{ij}(T,\dot{T})$ can be re-expressed as follows:

(9) $$A_{ij}=\left\{\begin{array}{c} k_{ij}(T)p_{i}-l_{ij}(T)p_{j}\\ if\;k_{ij}(T)p_{i}-l_{ij}(T)p_{j}>0(from\;i\;to\;j\;transf.)\\ -k_{ji}(T)p_{j}+l_{ji}(T)p_{i}\\ if\;k_{ji}(T)p_{j}-l_{ji}(T)p_{i}>0(from\;j\;to\;i\;transf.)\\ 0\;if\;k_{ij}(T)p_{i}-l_{ij}(T)p_{j}\leq 0\\ and\;k_{ji}(T)p_{j}-l_{ji}(T)p_{i}\leq 0\\ \left(no\;transformation\;between\;phases\;i\;and\;j\right) \end{array}\right.$$

where $k_{ij}$ and $l_{ij}$ are the parameters to be determined from the calibration with the CCT diagram. Following the suggestion in the reference of Leblond and Devaux [42], they are defined as follows:

(10) $$k_{ij}=\frac{P_{eq}(T)}{\tau },l_{ij}=\frac{1-P_{eq}(T)}{\tau }$$

where $P_{eq}(T)$ is the equilibrium phase volume proportion after an infinitely long time, which has a value between 0 and 1. τ is the characteristic time necessary for an equilibrium state. $P_{eq}(T)$ is determined by Equation (11) as follows:

(11) $$P_{eq}(T)=\frac{T_{s}-T}{T_{s}-T_{f}}$$

Note that the derivative of the phase can be defined as Equation (12)

(12) $$\frac{dP}{dt}=\frac{P_{eq}-P}{\tau }$$

From Taylor’s expansion to Equation (12), we have the phases $(P_{n+1}$) at the time $t_{n+1}$ as in Equation (13):

(13) $$P_{n+1}=P_{n}+{\left.\frac{dP}{dt}\right|}_{n}{\left(\frac{dT}{dt}\right)}^{-1}\Delta T=P_{n}+\frac{P_{eq}-P}{\tau }{\left(\frac{dT}{dt}\right)}^{-1}\Delta T$$

Calibration of the CCT diagram of DH36 steel was conducted for metallurgical analysis. In the present study, the CCT diagrams in the reference [48] were used for the calibration. Iterative computations of Equation (13) were performed by finding τ such that the temperature T_f_ was in good agreement with the one in the CCT diagram. Table 2 shows the metallurgical parameters, τ determined from the calibration for ferritic and bainitic transformations. Table 3, Table 4 and Table 5 show the start and finish temperatures for ferritic and bainitic transformations, which were obtained from the present study and the CCT diagram. The results show that the metallurgical analysis in the present study is in good agreement with the one in CCT diagram. The maximum differences in temperature from the two results are overall less than 5 °C.

### 2.3. Mechanical Analysis

A thermo-mechanical constitutive model incorporated with the transformation plasticity proposed by Leblond et al. [11,12] were implemented into the present numerical model. The multiple phases in steel were categorized into two phases: the hard phase (α-phase: ferrite, bainite, and martensite) and the weak phase (γ-phase: the austenite). As discussed above, Fe atoms in steel are arranged in either BCC or FCC structure depending on temperature. Ferritic steel has a BCC structure at room temperature, while austenite has a FCC structure above the eutectoid temperature. The terms of “hard” and “weak” are originated from the level of yield stress. The hard phase has the higher yield stress than the weak phase.

The total strain $\varepsilon^{tot}$ is composed of the elastic strain $\varepsilon^{el}$, the thermal strain $\varepsilon^{th}$, the strain due to transformation plasticity $\varepsilon^{tp}$, and the strain due to conventional plasticity $\varepsilon^{cp}$, as shown in Equation (14).

(14) $$\varepsilon^{tot}=\varepsilon^{el}+{\varepsilon^{th}+\varepsilon }^{tp}+\varepsilon^{cp}$$

The yield stress of the α-phase ($\sigma_{2}^{y}$) is calculated as an average of yield stresses of each phase as shown in Equation (15):

(15) $$\sigma_{2}^{y}(T)=\sum_{i}\;p_{i}\sigma_{i}^{y}$$

where $p_{i}$ denotes the volume proportion of each phase, while $\sigma_{i}^{y}$ is the yield stress of each phase i. The mixture rule is applied to determine the yield stress of multiple-phases and is given by:

(16) $$\sigma^{y}\left(\varepsilon_{1}^{eff},\varepsilon_{2}^{eff},T\right)=[1-f(z)]\sigma_{1}^{y}\left(T,\varepsilon_{1}^{eff}\right)+f(z)\sigma_{2}^{y}\left(T,\varepsilon_{2}^{eff}\right)$$

Here, $\sigma_{1}^{y}$ and $\sigma_{2}^{y}$, is the yield stress of the weak phase and the hard phase, respectively.

z is a summation of the phase volume fraction of the hard phases. f(z) is the modification factor [12] for the nonlinear mixture rule listed in Table 6.

Following the Leblond’s characterization of the flow rule [11,12], the plastic deformation in the present numerical model is categorized into the two types: the transformation plasticity and the conventional macroscopic plasticity.

First, the deformation is assumed to be a purely elastic and the trial stress is calculated as follows:

(17) $$\sigma_{n+1}^{\mathrm{trial}\;}=\sigma_{n}+C^{el}:\Delta {\hat{\varepsilon }}^{RN}+\Delta C:\varepsilon_{n}^{el}$$

where $\sigma_{n+1}^{\mathrm{trial}\;}$ is the trial stress at the current time step; $C^{el}$ is the elastic tangent moduli; $\Delta {\hat{\varepsilon }}^{RN}$ is the rotation-neutralized strain increment; and $\varepsilon_{n}^{el}$ is the elastic strain at the previous time step. Once the trial stress is updated, it is compared with the yield stress to determine whether a material undergoes the elastic or plastic deformation. If the deformation turns out to be elastic, the final stress is updated with the trial stress and the simulation moves to the next time step.

**Figure 1:**
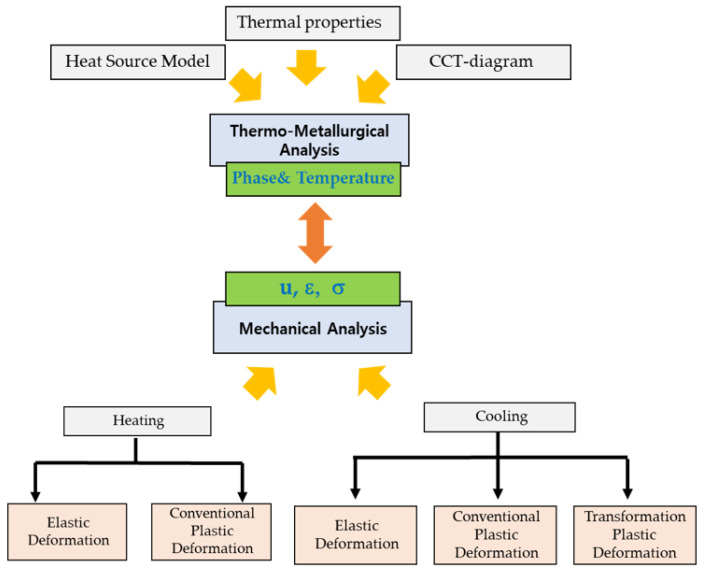
The schematic of the present numerical model.

The plastic deformation is categorized into two types as described above: the transformation plasticity ($\bar{\sigma }\leq \sigma^{y}$, in cooling) and the conventional macroscopic plasticity. For the stress-update of the plastic deformation, the stress relaxation is conducted using the radial-return scheme in a different way, depending on the type of plasticity (Equations (18)–(22) for the transformation plasticity; Equations (23)–(26) for the conventional macroscopic plasticity). The overall schematic of the present numerical model is demonstrated in Figure 1.

Case 1: Transformation plasticity ($\bar{\sigma }\leq \sigma^{y}$)

(18) $${\dot{\varepsilon }}^{p}=\frac{3}{2}\frac{{\dot{\bar{\varepsilon }}}^{p}}{\bar{\sigma }}s=\sqrt{\frac{3}{2}}n\;\mathrm{with}\;n=\sqrt{\frac{3}{2}}\frac{s}{\bar{\sigma }}$$

(19) $${\dot{\bar{\varepsilon }}}^{p}=\sqrt{\frac{3}{2}}\frac{1-z}{\sigma_{1}^{y}\left({\bar{\varepsilon }}_{1}^{\mathrm{eff}\;}\right)}{\dot{\varepsilon }}_{1}^{eff}∥s∥$$

(20) $$\begin{array}{cc} {\dot{{\bar{\varepsilon }}_{1}}}^{eff}= & \frac{-2\Delta \varepsilon_{1\to 2}^{th}}{1-z}(lnz)\dot{z}\hat{h}\left(∥s∥,{\dot{\varepsilon }}_{1}^{eff},{\dot{\varepsilon }}_{2}^{eff}\right)+\sqrt{\frac{3}{2}}\frac{g(z)}{E}∥s∥\\ & +\frac{2\left(\alpha_{1}-\alpha_{2}\right)zlnz}{1-z}\dot{T} \end{array}$$

(21) $${\dot{\bar{\varepsilon }}}_{2}^{eff}=-\frac{\dot{z}}{z}{\bar{\varepsilon }}_{2}^{eff}+\omega^{\dot{z}}{\bar{\varepsilon }}_{1}^{eff}$$

(22) $$\bar{\sigma }=\sqrt{\frac{3}{2}s:s}$$

where s is the deviatoric stress; $\Delta \varepsilon_{1\to 2}^{th}$ is the difference in thermal strain between the α- and β-phases; $\hat{h}\left(∥s∥,{\dot{\varepsilon }}_{1}^{eff},{\dot{\varepsilon }}_{2}^{eff}\right)$ is a function that takes into account nonlinearities in the stress; E is the Young’s modulus; $\alpha_{1}$ and $\alpha_{2}$ are the coefficients of thermal expansion of the weak and hard phases, respectively. g(z) (listed in Table 6) is a modification function for small values of z. Leblond and Devaux [12] obtained the values of g(z) from the curve fitting on the stress–strain data. Such values are used in the present study and listed in Table 6.

Case 2: Conventional macroscopic plasticity ($\bar{\sigma }=\sigma^{y}$)

(23) $${\dot{\varepsilon }}^{p}=\frac{3}{2}\frac{{\dot{\bar{\varepsilon }}}^{p}}{\bar{\sigma }}s$$

(24) $$\bar{\sigma }=\sigma^{y}$$

(25) $${\dot{\bar{\varepsilon }}}_{1}^{eff}={\dot{\bar{\varepsilon }}}^{p}$$

(26) $${\dot{\bar{\varepsilon }}}_{2}^{eff}={\dot{\bar{\varepsilon }}}_{2}^{p}-\frac{\dot{z}}{z}{\bar{\varepsilon }}_{2}^{eff}+\omega \frac{\dot{z}}{z}{\bar{\varepsilon }}_{1}^{eff}$$

### 2.4. Finite Element Model

**Figure 2:**
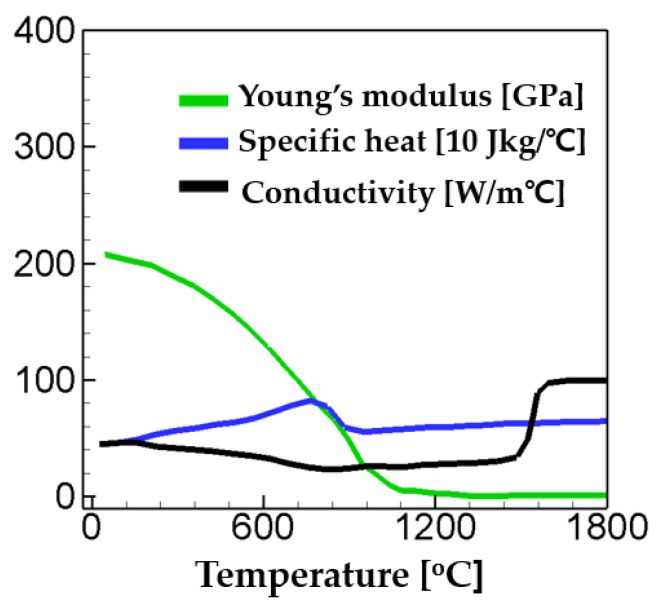
Material properties used in the present study (adapted from [47]).

Figure 2 shows the material properties used in the present study. The values of the material properties were taken from [47] and plotted in Figure 2. The yield stresses of each phase are listed in Table 7. The values of thermal expansion coefficients of the weak and the hard phase ($\varepsilon_{1}=$ 2.260 × 10^−5^ °C^−1^; $\varepsilon_{2}=$ 1.615 × 10^−5^ °C^−1^) and the Poisson’s ratio (ν = 0.3) were employed from [48]. In the present work, four different welding speeds (v = 25, 35, 40, 60 cm/min) are considered to investigate the influence of welding speed on the residual stress generation. The simulations were performed with and without consideration of the phase transformation for each welding speed. Therefore, total eight simulation cases were conducted for analysis of thermal profiles and residual stress. The simulation cases considered in the present study are listed in Table 8.

The numerical model discussed in Section 2.1, Section 2.2 and Section 2.3 were implemented into the ABAQUS user-subroutines UMAT, UMATHT, DFLUX, and UEXPAN. In the UEXPAN, the thermal strain of the multiple-phase steel is calculated. In DFLUX, the heat source is defined including the size, position, velocity, and the level of heat energy. The thermal behaviors of a material with an account of the phase transformation are defined in the UMATHT. The calculation of the phase volume fraction and the stress-update are conducted in the UMAT subroutine.

**Figure 3:**
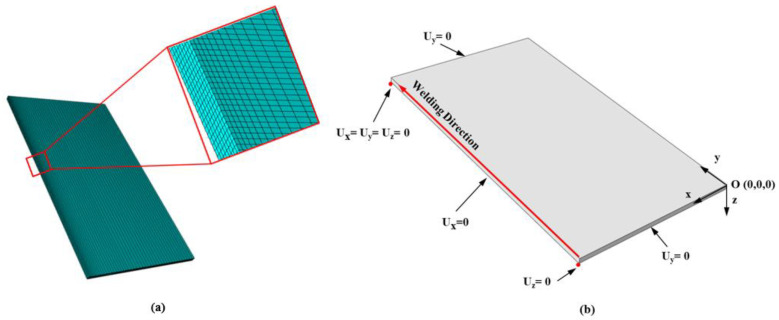
(**a**) Finite element model; (**b**) boundary conditions for the present numerical study.

In this work, the dimensions of the FE model are 1100 mm × 600 mm × 12.5 mm. A half symmetry condition was imposed to reduce the computation time, and some of nodes were constrained to prevent the rigid body motion as shown in Figure 3b. The local mesh refinement was applied to the fusion zone (FZ) and heat-affected zone (HAZ) with their minimum size of about 1 mm to optimize the accuracy and efficiency of the FE simulation. Such meshing was determined based on the preliminary simulations which were performed to identify the region where mesh refinement is required. The model was discretized with 58,264 C3D8T elements (8-node trilinear element for thermo-mechanically fully coupled analysis). There could be other options for the choice of element, for example, (1) sequentially coupled elements, or (2) 2D elements. However, C3D8T element was chosen in the present study because it gives the better results with reasonable computation time than other options. The welding conditions in this work is 30.9 V, 1170A for the arc voltage and welding current, respectively. The total computation time was set to 4000 s for the model to reach the room temperature after welding. The time step was chosen as the automatic time increment so that the size of the time step can be adjustable based on the solution convergence rates.

## 3. Results and Discussion

**Figure 4:**
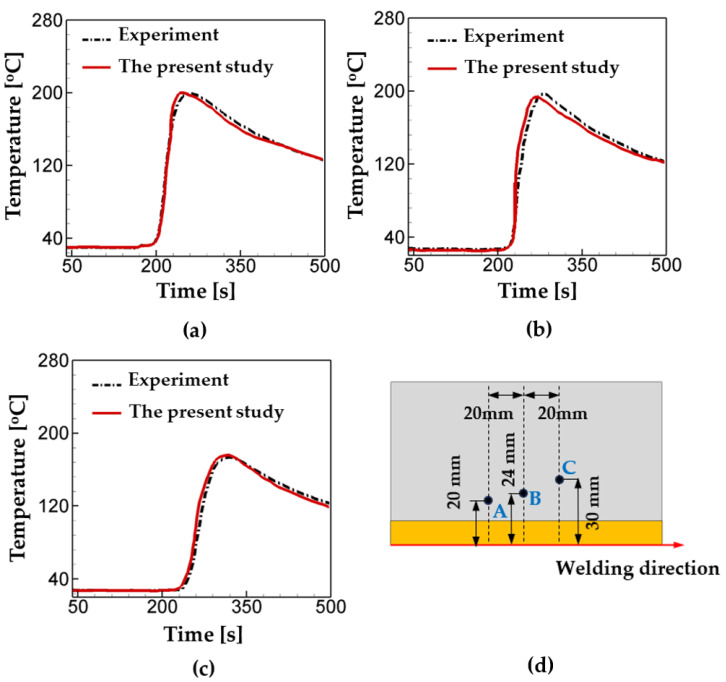
Comparison of the temperature calculations from the present model with the thermocouple measurements at the different locations: (**a**) Point A, (**b**) Point B, (**c**) Point C, and (**d**) the locations of thermocouple arrangement.

First, for verification of thermal analysis in the present numerical model, the values of temperature calculated from the simulations are compared with thermocouple measurements from Fu et al. [47]. Following the experiment set-up, welding speed, welding current, and voltage are set to be 560 A, 30 V, and 27 cm/min, and three points of investigation are selected. The comparison results are plotted in Figure 4 and listed in Table 9. As shown, the temperature history curves from the measurements and the simulations are good agreement with each other with the maximum error of 21.6%.

**Figure 5:**
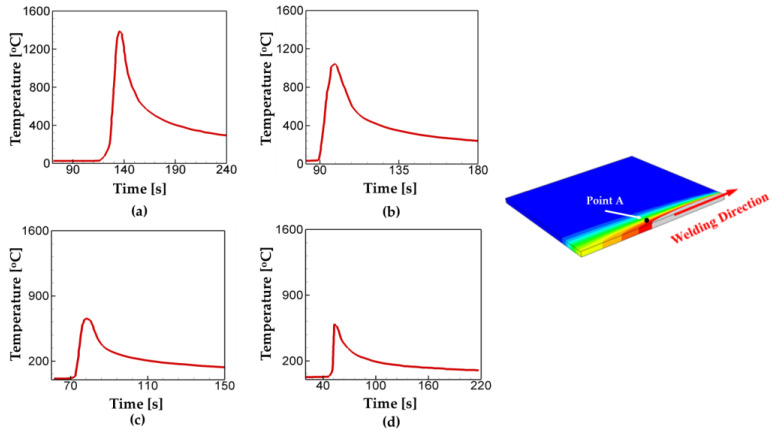
Temperature evolution at the point A for the different welding speed: (**a**) case 1, (**b**) case 2, (**c**) case 3, and (**d**) case 4. The point A locates 8 mm away from the welding line. Details on the welding conditions are listed in Table 8.

Next, the effects of welding speed on thermal profiles are investigated for the cases listed in Table 8. For such an investigation, the point A was chosen, which was located 8 mm away from the center of the welding line, as shown in Figure 5. Hereafter, this point is named as point A. Figure 5 shows the temperature evolution for the different welding speed and the location of the point A. As shown in Figure 5, the peak temperature decreases as welding speed increases. While the peak temperature for case 1 is calculated as T = 1391 °C, the values for cases 2, 3, 4 are T = 1044 °C, T = 764 °C, T = 596 °C, respectively. In case of the cooling rate from the peak temperature to T = 300 °C, it is found that case 4 (30.3 °C/s) has the highest cooling rate, while case 1 has the lowest one (12.1 °C/s) among the cases considered. The results suggest that the slow welding speed allows more heat accumulation and leads to a lower cooling rate.

**Figure 6:**
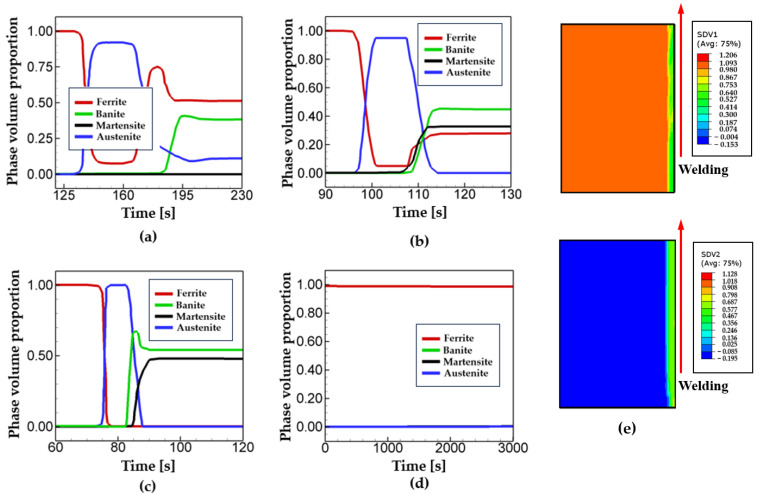
Phase evolution at the point A for the different welding speed: (**a**) case 1, (**b**) case 2, (**c**) case 3, (**d**) case 4, and (**e**) the phase fraction contours of ferrite and bainite at the end of the simulation for case 1.

Figure 6 shows the phase fraction at the Point A for different welding speed. The evolution of the microstructures in welding is significantly affected by thermal profiles. As described above, iron atoms in any steel are in a BCC structure at room temperature. When temperature exceeds the eutectoid temperature in heating process, the ferritic steel starts to transform into the austenitic steel, having a FCC atomistic structure. In cooling, austenite transforms into the ferrite, bainite, and martensite, depending on the thermal profiles. Analysis of Figure 5 and Figure 6 shows that the time for temperature to reach the eutectoid temperature (T = 741 °C [32]) coincides with the ones when austenite starts to be formed in heating process for cases 1~3. As shown in Figure 5, temperature history reaches the eutectoid temperature at t = 130 s, t = 96 s, and t = 75 s for cases 1~3. Such values are in overall good agreement with the times when phase transformation starts for each case as shown in Figure 6. Note that in Figure 5d, the peak temperature for case 4 is calculated as T = 596 °C, which means it does not pass the critical eutectoid temperature. This explains why phase transformation does not occur at the Point A for case 4 as shown in Figure 6d. In cooling, austenite starts to transform back into ferritic steel depending on thermal profiles such as the peak temperature, cooling rate, and temperature range. For case 1, the final phase volume fractions of ferrite and bainite are calculated as p_1_ = 0.51 and p_2_ = 0.38, respectively. For case 2, the values of ferrite, bainite, martensite are calculated as p_1_ = 0.26, p_2_ = 0.44 and p_3_ = 0.30, respectively. During the cooling process of welding, carbon atoms hardly diffuse out of the ferrous cell and trapped in the cell. Therefore, they are distorted and form a martensite phase. Differently from case 1, the cooling rates of case 2 (i.e., 24.2 °C/s) and case 3 (i.e., 28.3 °C/s) are high enough to nucleate and grow martensite phase, leading to the final phase fraction of p_3_ = 0.30 for case 2 and p_3_ = 0.48 for case 3. The results show that the phase fraction of martensite increases with an increase in cooling rate. Such a finding can be also found in other research (for example, Ravikumar et al. [49]). Figure 6e shows the phase fraction contours of ferrite and bainite at the end of the simulation for case 1, showing the heat-affected zone (HAZ) near the welding line. The phase fractions in Figure 6 show the consistency with other microstructure analyses of DH36 welding (for example, refs. [50,51]). Toumpis et al. [50] reported that the slow speed welding (10 cm/min) resulted in ferrite-dominant and homogeneous microstructures with refined grains; in the case of the intermediate welding speed (25 cm/mm), it was found that the microstructure consisted of acicular shaped bainite regions and ferrite-dominant regions; For the fast welding (50 cm/min), heterogeneous microstructures were exhibited with predominant bainite. It was reported that the phase fractions of bainite increases considerably with the cooling rate. Reynolds et al. [51] reported that bainitic and martensitic microstructures were formed at the welding speed of 45 cm/min.

In this work, the effects of welding speed on thermal profiles, and residual stress are investigated. For such investigation, simulations were performed with and without considering the phase transformation for the different welding speed. All cases considered in the present study are listed in Table 8. In Table 8, ‘sp’ (single phase) cases indicate the simulations neglecting phase transformation, and thus, the elastic and conventional plasticity are only considered. On the contrary, the ‘mp’ (multiple phases) cases are the simulations considering the phase transformation and transformation plasticity. In these cases, the elastic, conventional plastic, and transformation plastic deformation are considered as described in Section 2.3.

**Figure 7:**
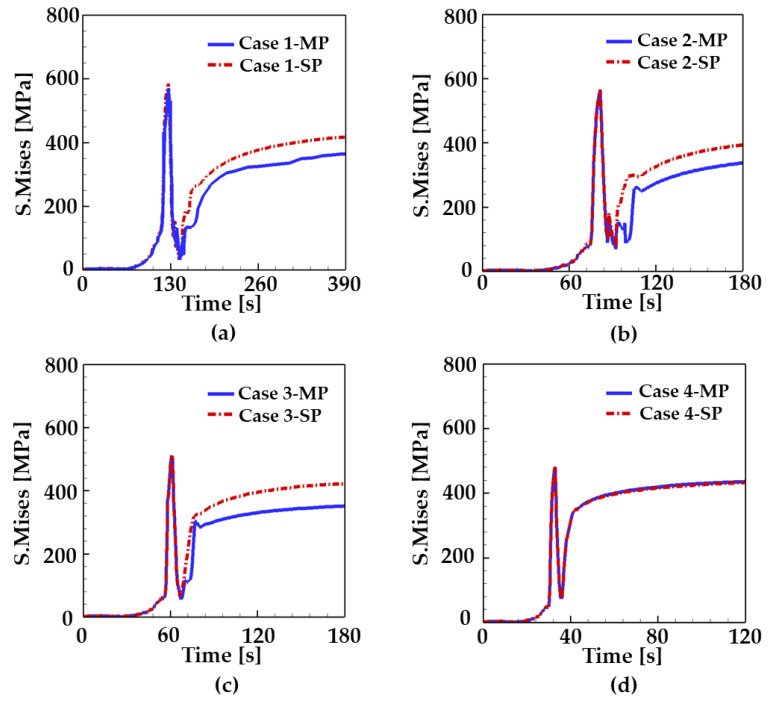
Mises stress as a function of time for different welding speed at the point A. (**a**) case 1, (**b**) case 2, (**c**) case 3, and (**d**) case 4. Refer to Table 8 for details on the simulation conditions.

Figure 7 shows the Mises stress history at the Point A for the different welding speed. First, analyses show that the residual stress increase with a decrease in the welding speed. This is mainly because the welding with lower speed allows more time for heat accumulation to the weldment. This eventually leads to the higher peak temperature and slow cooling rate which result in the ferrite-dominant microstructures. It is worth noting in Figure 5 and Figure 6 that the phase portion of martensite increase with the welding speed due to its high cooling rate. These findings support the trend of Mises stress curves in Figure 7.

Another interesting result of Figure 7 is that there exists a clear difference in Mises stress between the sp and mp cases. In other words, the values of Mises stress for the mp cases are lower than the ones for the sp cases for each welding speed. Such a difference results from the volume dilation between coexisting phases of steel. In the heating process, a compressive stress is generated in the FZ and HAZ. As heat continues to be applied to the weldment, the compressive stress gets smaller because the yield stress decreases at high temperatures. When heating is completed and cooling starts, the tensile stress is generated because of the shrinkage of the weldment [44]. However, when temperature reaches in the range of phase transformation (787–545 °C), the compressive stress is generated again due to the volume difference between the coexisting phases (i.e., transformation plasticity). Once the phase transformation is completed and the phase volume fraction is finalized, the amount of compressive stress produced due to multiple phases also remains constant. In Figure 7, the difference in Mises stress between the sp and mp cases for each welding speed becomes constant at t = 210 s, t = 115 s, and t = 90 s for case 1, 2, and 3, respectively. Such values of time are good agreement with the ones when the phase transformation is completed and the phase volume fraction is finalized in Figure 6. The same trends were also observed in other studies in the literatures. For example, Cho and Kim [52] performed the simulations and experiments of welding to investigate the effects of martensite transformation on the residual stress generation in AISI 1045 and AISI 1020 steels. In their experiments, the compressive stress was observed in the weld zone, which was produced due to volume change from phase transformation. Tajat et al. [53] measured the welding-induced residual stress using the neutron diffraction technique. The results showed that the longitudinal stress in the FZ and HAZ is lower than the one in base material adjacent to the HAZ. However, it is worth to noting that there exists a variation in thermal expansion at the interface between the HAZ and base metal due to their different microstructural features. Such variation results in the tensile stress near the interface as reported in previous studies (e.g., refs. [54,55]). This is detrimental to the fatigue life of the components. Note that in the case of a welding speed of v = 60 cm/min (i.e., case 4), no phase transformation occurs because its peak temperature does not reach the eutectoid temperature, as shown in Figure 7d. This explains why the Mises stress curves for case 4-mp and case 4-sp coincide each other.

**Figure 8:**
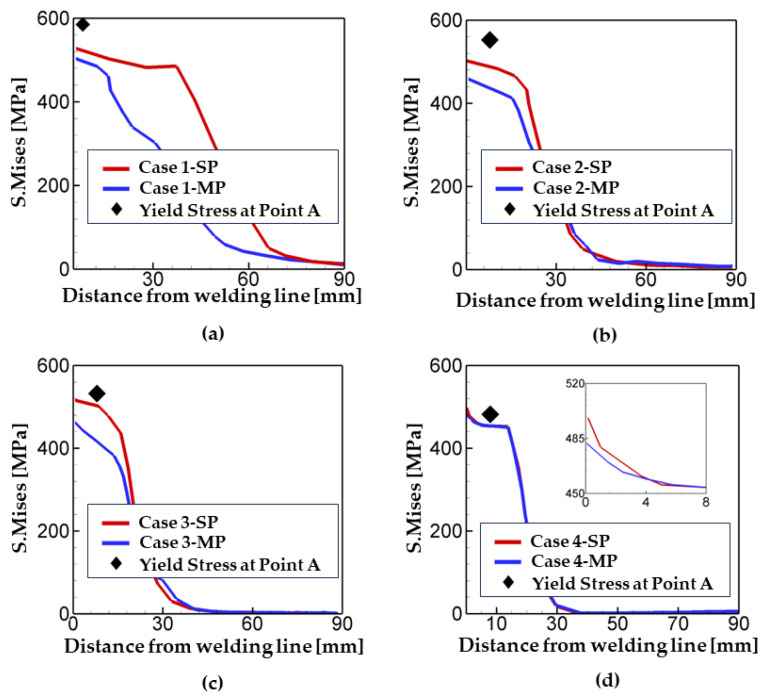
Mises stress as a function of distance from the welding line for different welding speed. (**a**) case 1, (**b**) case 2, (**c**) case 3, and (**d**) case 4. Refer to Figure 5 for the location of point A.

In order to analyze the influence of welding speed on the residual stress generation, the values of Mises stress for the sp and mp cases are compared and plotted as a function of the distance from welding line as shown in Figure 8 along with the yield stress of a welded steel at the point A. Note that the yield stress is various depending on the cooling rate. The point A was chosen for the comparison. In Figure 8, the zone where a clear difference between the sp and mp cases is observed is the HAZ. Figure 8 shows that the width of HAZ decreases with an increase in welding speed, and thus, the effect of phase transformation on the residual stress generation in a weldment is also reduced. For case 1 (welding speed = 25 cm/min), the width of HAZ is calculated as 66 mm, while it decreases to 18 mm in case of the welding speed = 40 cm/min (i.e., case 3). If the welding speed is greater than 60 cm/min, the width of HAZ becomes very narrow and there is little effect of phase transformation on residual stress, as shown in Figure 8d.

## 4. Conclusions

In this work, a thermo-mechanical constitutive model considering the phase transformation and transformation plasticity was implemented into the numerical model by way of ABAQUS user-subroutines. In order to consider the phase evolution in welding, the metallurgical parameters for the Leblond’s phase equation were obtained from the calibration with a CCT diagram of DH36 steel. In addition, the effects of welding speed on the thermal profiles and residual stress generation were investigated. Major findings are the following:

1. The results show that residual stress increases with a decrease in welding speed. Lower speed of welding allows more time for heat accumulation to a weldment. This leads to the higher peak temperature and slow cooling rate which result in the ferrite-dominant microstructures.
2. The width of HAZ decreases as the welding speed increases. It is shown from the numerical results with and without consideration of phase transformation that the phase volume fraction is significantly affected by the welding speed. Such phase transformation leads to the generation of a compressive stress in the FZ and HAZ.
3. Simulation results show that the phase fraction of martensite increases with a welding speed because of its high cooling rate. The volume difference between the coexisting phases produces a compressive stress in cooling and its magnitude increases with an increase in martensite phase fraction.
4. Since the thermal profiles such as cooling rate and peak temperature are significantly affected by the welding speed, phase transformation should be considered for accurate analysis of residual stress in a weldment.

Residual stress generation is one of the major concerns in welding. Such a challenge mainly results from the rapid heating and cooling during welding. The present numerical model allows the multiphysics simulation which is coupled with thermal, mechanical, and metallurgical behaviors of a material. The present work can be extended to the welding of different materials such as aluminum and titanium alloys. An investigation on the welding process parameters such as interpass idle time and welding trajectory for multipass welding is underway to establish the optimal welding conditions.

## Figures and Tables

**Table 1:** Goldak’s heat source parameters (taken from [47]).

$a_{f}$ (mm)	$a_{r}$ (mm)	b (mm)	c (mm)	$f_{f}$	$f_{r}$
20	80	20	22	0.4	1.6

**Table 2:** The parameter (τ) for metallurgical analysis in the present numerical model.

Cooling Rate [°C/s]	Peak Temperature [°C]	τ [s] (Ferritic Transformation)	τ [s] (Bainitic Transformation)
1	875	0.92	0.0002
1	1000	0.94	0.0004
1	1350	0.001	0.005
5	875	0.02	0.012
5	1150	0.003	0.004
5	1350	0.96	0.0231
10	875	1.05	0.0154
10	1350	1.26	0.024
25	875	1.45	0.0369
25	1350	1.654	0.005
100	875	1.032	0.005
100	1000	1.727	0.027
200	875	1.234	0.0031
200	1000	1.256	0.0284

**Table 3:** Comparison of the start temperature calculations for ferritic transformation from the present simulations with the ones in the CCT diagram as a function of cooling rate and peak temperature.

Cooling Rate [°C/s]	Peak Temperature [°C]	CCT [°C]	Present Result [°C]
1	875	787	783
1	1000	788	785
1	1150	752	754
1	1350	768	771
5	875	755	752
5	1000	750	750
5	1150	700	703
5	1350	689	692
10	875	762	759
10	1000	744	747
10	1150	694	692
10	1350	660	665
25	875	788	782
25	1000	717	715
25	1150	682	680
25	1350	627	620
100	875	678	673
100	1000	704	708
200	875	682	685
200	1000	689	692

**Table 4:** Comparison of the finish temperature calculations for ferritic transformation from the present simulations with the ones in the CCT diagram as a function of cooling rate and peak temperature.

Cooling Rate [°C/s]	Peak Temperature [°C]	CCT [°C]	Present Result [°C]
1	875	674	669
1	1000	672	674
1	1150	596	592
1	1350	572	578
5	875	657	655
5	1000	658	654
5	1150	545	550
5	1350	564	559
10	875	643	642
10	1000	649	643
10	1150	546	542
10	1350	521	520
25	875	607	609
25	1000	585	592

**Table 5:** Comparison of the start temperature calculations for bainitic transformation from the present simulations with the ones in the CCT diagram as a function of cooling rate and peak temperature.

Cooling Rate [°C/s]	Peak Temperature [°C]	CCT [°C]	Present Result [°C]
100	875	674	669
100	1000	672	674
100	1150	596	592
200	1350	572	578
200	875	657	655
200	1000	658	654

**Table 6:** Values of functions f(z) and g(z) (taken from [12]).

z	f(z)	g(z)
0	0	0
0.125	0.044	2.53
0.25	0.125	4
0.5	0.391	2.76
0.75	0.668	1.33
1.0	1	1

**Table 7:** Yield stresses of each phase (taken from [48]).

Temperature [°C]	Ferrite [MPa]	Bainite [MPa]	Martensite [MPa]	Austenite [MPa]
20	425	405	750	220
700	110	100	210	98
1300	12.5	12.5	12.5	12.5

**Table 8:** Simulation cases considered in the present study.

Case No.	Welding Speed [cm/min]	Consideration of Phase Transformation
Case 1-SP	25	No
Case 1-MP	25	Yes
Case 2-SP	35	No
Case 2-MP	35	Yes
Case 3-SP	40	No
Case 3-MP	40	Yes
Case 4-SP	60	No
Case 4-MP	60	Yes

**Table 9:** Comparison of the temperature calculations from the present model with the thermocouple measurements at the different locations: The points of investigation are illustrated in Figure 4d.

Time [s]	Point A			Point B			Point B		
	Num [°C]	Exp [°C]	Error [%]	Num [°C]	Exp [°C]	Error [%]	Num [°C]	Exp [°C]	Error [%]
200	38.2	38.5	0.8	25.6	26.9	5.1	27.6	27.4	1.0
220	126.1	133.3	5.7	30.0	30.2	0.8	27.8	28.1	1.0
240	199.4	191.2	4.1	145.8	114.3	21.6	40.3	33.7	16.4
260	197.6	199.7	1.1	191.7	179.2	6.5	96.5	78.4	18.7
280	191.7	194.5	1.5	188.5	197.1	4.6	151.1	138.4	8.4
300	183.1	186.2	1.7	181.3	189.0	4.3	173.6	168.5	2.9
320	170.4	177.0	3.9	172.0	180.5	4.9	175.2	173.4	1.0
340	161.8	165.5	2.3	165.8	172.3	3.9	167.9	169.1	0.7
360	155.9	162.1	4.0	156.3	161.2	3.1	159.8	160.8	0.7
380	149.1	153.0	2.6	146.7	152.8	4.2	150.5	154.1	2.4
400	145.6	148.3	1.9	144.5	148.9	3.1	142.7	147.2	3.1
420	142.2	141.9	0.2	137.7	142.6	3.6	136.4	140.0	2.6
440	139.2	139.8	0.4	131.1	134.6	2.7	132.6	135.2	2.1
460	134.7	133.0	1.2	127.8	130.5	2.1	125.3	130.6	4.2
480	130.5	129.9	0.5	124.7	125.8	0.9	119.0	127.4	7.1
500	125.3	125.4	0.1	122.0	123.1	0.9	118.2	123.2	3.6

## Data Availability

The data that support the findings of this study are available from the corresponding author upon request.
